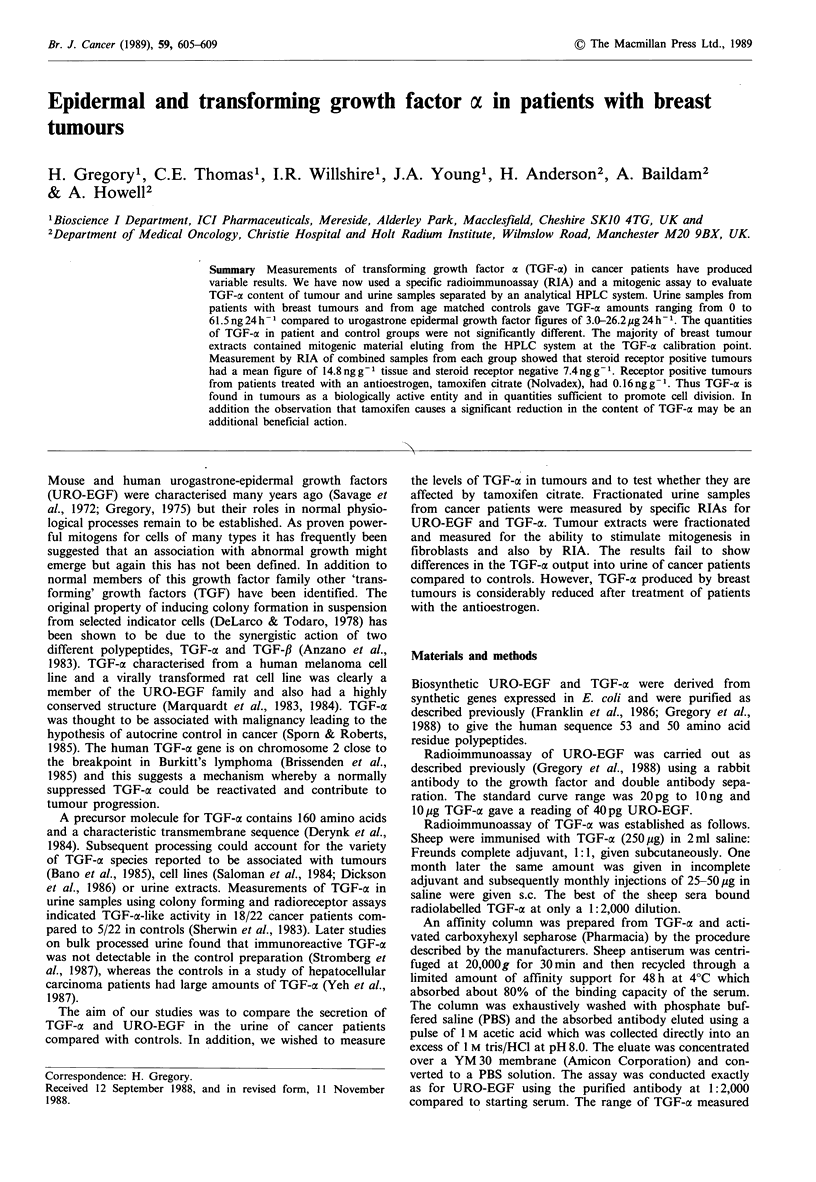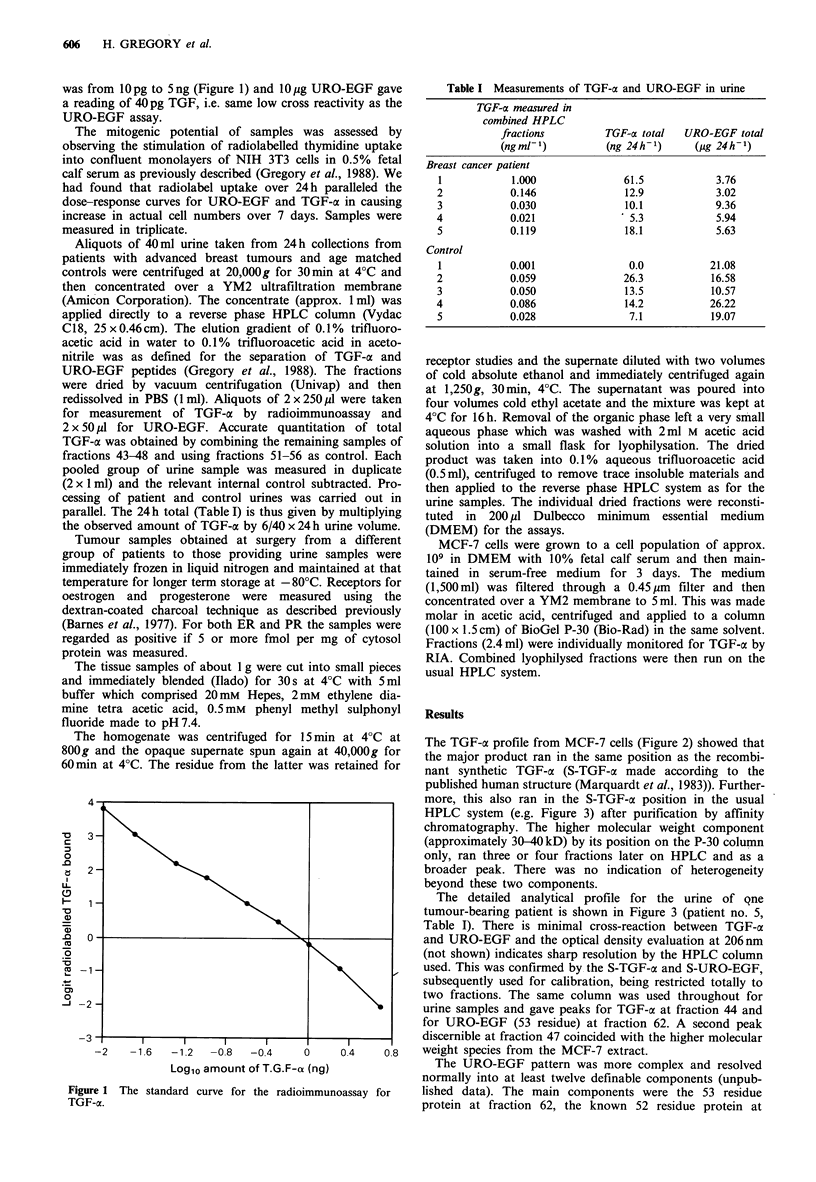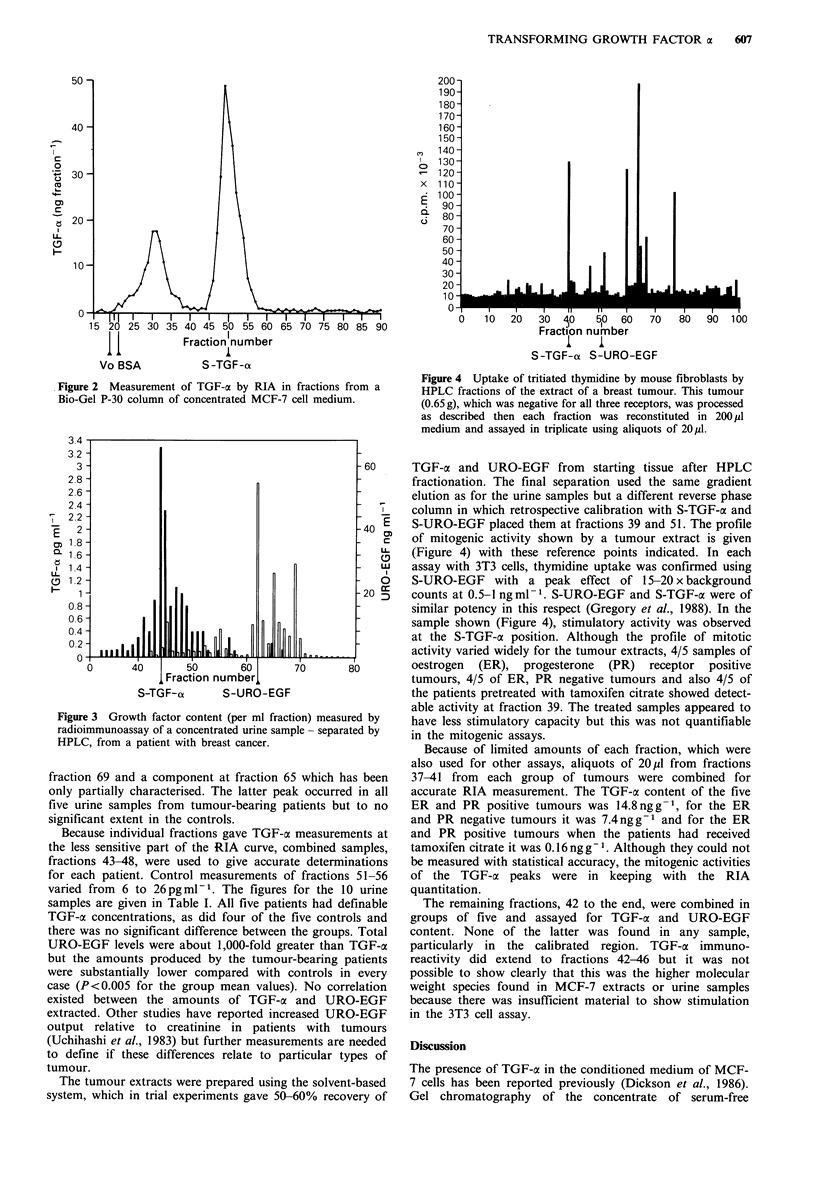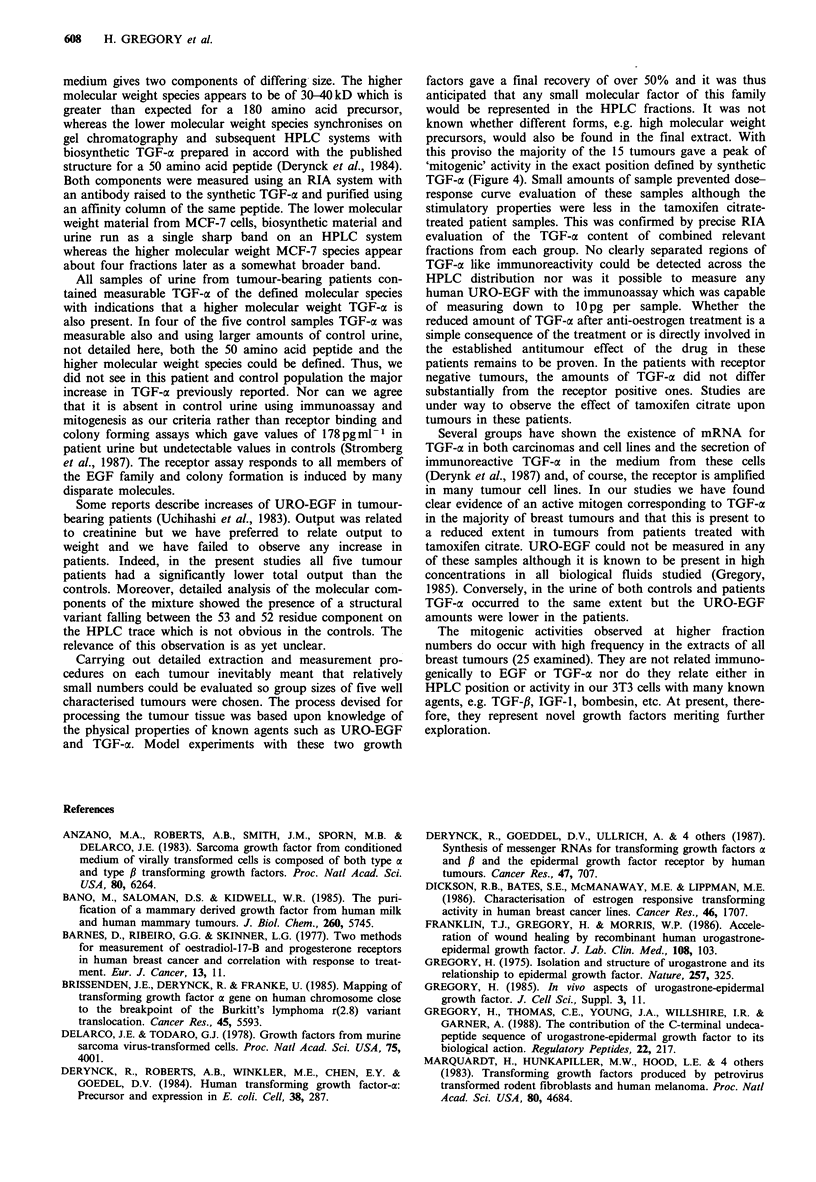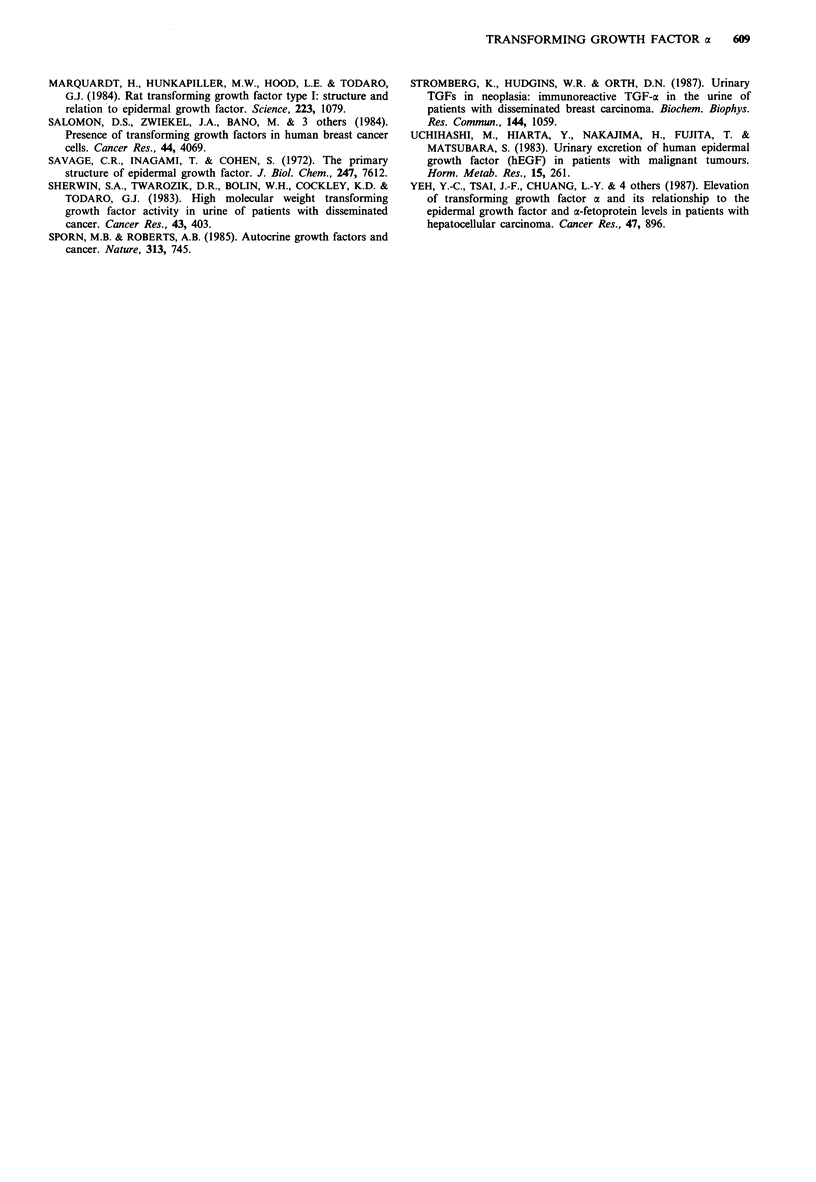# Epidermal and transforming growth factor alpha in patients with breast tumours.

**DOI:** 10.1038/bjc.1989.122

**Published:** 1989-04

**Authors:** H. Gregory, C. E. Thomas, I. R. Willshire, J. A. Young, H. Anderson, A. Baildam, A. Howell

**Affiliations:** Bioscience I Department, ICI Pharmaceuticals, Macclesfield, Cheshire, UK.

## Abstract

Measurements of transforming growth factor alpha (TGF-alpha) in cancer patients have produced variable results. We have now used a specific radioimmunoassay (RIA) and a mitogenic assay to evaluate TGF-alpha content of tumour and urine samples separated by an analytical HPLC system. Urine samples from patients with breast tumours and from age matched controls gave TGF-alpha amounts ranging from 0 to 61.5 ng 24 h-1 compared to urogastrone epidermal growth factor figures of 3.0-26.2 micrograms 24 h-1. The quantities of TGF-alpha in patient and control groups were not significantly different. The majority of breast tumour extracts contained mitogenic material eluting from the HPLC system at the TGF-alpha calibration point. Measurement by RIA of combined samples from each group showed that steroid receptor positive tumours had a mean figure of 14.8 ng g-1 tissue and steroid receptor negative 7.4 ng g-1. Receptor positive tumours from patients treated with an antioestrogen, tamoxifen citrate (Nolvadex), had 0.16 ng g-1. Thus TGF-alpha is found in tumours as a biologically active entity and in quantities sufficient to promote cell division. In addition the observation that tamoxifen causes a significant reduction in the content of TGF-alpha may be an additional beneficial action.


Br. J. Cancer (1989), 59, 605–609 © The Macmillan Press Ltd., 1989

### Epidermal and transforming growth factor α in patients with breast tumours

H. Gregory[1], C.E. Thomas[1], I.R. Willshire[1], J.A. Young[1], H. Anderson[2], A. Baildam[2] & A. Howell[2]

[1]*Bioscience I Department, ICI Pharmaceuticals, Mereside, Alderley Park, Macclesfield, Cheshire SK10 4TG, UK and* [2]*Department of Medical Oncology, Christie Hospital and Holt Radium Institute, Wilmslow Road, Manchester M20 9BX, UK.*

**Summary** Measurements of transforming growth factor α (TGF-α) in cancer patients have produced variable results. We have now used a specific radioimmunoassay (RIA) and a mitogenic assay to evaluate TGF-α content of tumour and urine samples separated by an analytical HPLC system. Urine samples from patients with breast tumours and from age matched controls gave TGF-α amounts ranging from 0 to 61.5 ng 24 h$^{-1}$ compared to urogastrone epidermal growth factor figures of 3.0–26.2 μg 24 h$^{-1}$. The quantities of TGF-α in patient and control groups were not significantly different. The majority of breast tumour extracts contained mitogenic material eluting from the HPLC system at the TGF-α calibration point. Measurement by RIA of combined samples from each group showed that steroid receptor positive tumours had a mean figure of 14.8 ng g$^{-1}$ tissue and steroid receptor negative 7.4 ng g$^{-1}$. Receptor positive tumours from patients treated with an antioestrogen, tamoxifen citrate (Nolvadex), had 0.16 ng g$^{-1}$. Thus TGF-α is found in tumours as a biologically active entity and in quantities sufficient to promote cell division. In addition the observation that tamoxifen causes a significant reduction in the content of TGF-α may be an additional beneficial action.

Mouse and human urogastrone-epidermal growth factors (URO-EGF) were characterised many years ago (Savage *et al.*, 1972; Gregory, 1975) but their roles in normal physiological processes remain to be established. As proven powerful mitogens for cells of many types it has frequently been suggested that an association with abnormal growth might emerge but again this has not been defined. In addition to normal members of this growth factor family other 'transforming' growth factors (TGF) have been identified. The original property of inducing colony formation in suspension from selected indicator cells (DeLarco & Todaro, 1978) has been shown to be due to the synergistic action of two different polypeptides, TGF-α and TGF-β (Anzano *et al.*, 1983). TGF-α characterised from a human melanoma cell line and a virally transformed rat cell line was clearly a member of the URO-EGF family and also had a highly conserved structure (Marquardt *et al.*, 1983, 1984). TGF-α was thought to be associated with malignancy leading to the hypothesis of autocrine control in cancer (Sporn & Roberts, 1985). The human TGF-α gene is on chromosome 2 close to the breakpoint in Burkitt's lymphoma (Brissenden *et al.*, 1985) and this suggests a mechanism whereby a normally suppressed TGF-α could be reactivated and contribute to tumour progression.

A precursor molecule for TGF-α contains 160 amino acids and a characteristic transmembrane sequence (Derynk *et al.*, 1984). Subsequent processing could account for the variety of TGF-α species reported to be associated with tumours (Bano *et al.*, 1985), cell lines (Saloman *et al.*, 1984; Dickson *et al.*, 1986) or urine extracts. Measurements of TGF-α in urine samples using colony forming and radioreceptor assays indicated TGF-α-like activity in 18/22 cancer patients compared to 5/22 in controls (Sherwin *et al.*, 1983). Later studies on bulk processed urine found that immunoreactive TGF-α was not detectable in the control preparation (Stromberg *et al.*, 1987), whereas the controls in a study of hepatocellular carcinoma patients had large amounts of TGF-α (Yeh *et al.*, 1987).

The aim of our studies was to compare the secretion of TGF-α and URO-EGF in the urine of cancer patients compared with controls. In addition, we wished to measure the levels of TGF-α in tumours and to test whether they are affected by tamoxifen citrate. Fractionated urine samples from cancer patients were measured by specific RIAs for URO-EGF and TGF-α. Tumour extracts were fractionated and measured for the ability to stimulate mitogenesis in fibroblasts and also by RIA. The results fail to show differences in the TGF-α output into urine of cancer patients compared to controls. However, TGF-α produced by breast tumours is considerably reduced after treatment of patients with the antioestrogen.

### Materials and methods

Biosynthetic URO-EGF and TGF-α were derived from synthetic genes expressed in *E. coli* and were purified as described previously (Franklin *et al.*, 1986; Gregory *et al.*, 1988) to give the human sequence 53 and 50 amino acid residue polypeptides.

Radioimmunoassay of URO-EGF was carried out as described previously (Gregory *et al.*, 1988) using a rabbit antibody to the growth factor and double antibody separation. The standard curve range was 20 pg to 10 ng and 10 μg TGF-α gave a reading of 40 pg URO-EGF.

Radioimmunoassay of TGF-α was established as follows. Sheep were immunised with TGF-α (250 μg) in 2 ml saline: Freunds complete adjuvant, 1:1, given subcutaneously. One month later the same amount was given in incomplete adjuvant and subsequently monthly injections of 25–50 μg in saline were given s.c. The best of the sheep sera bound radiolabelled TGF-α at only a 1:2,000 dilution.

An affinity column was prepared from TGF-α and activated carboxyhexyl sepharose (Pharmacia) by the procedure described by the manufacturers. Sheep antiserum was centrifuged at 20,000 *g* for 30 min and then recycled through a limited amount of affinity support for 48 h at 4°C which absorbed about 80% of the binding capacity of the serum. The column was exhaustively washed with phosphate buffered saline (PBS) and the absorbed antibody eluted using a pulse of 1 M acetic acid which was collected directly into an excess of 1 M tris/HCl at pH 8.0. The eluate was concentrated over a YM 30 membrane (Amicon Corporation) and converted to a PBS solution. The assay was conducted exactly as for URO-EGF using the purified antibody at 1:2,000 compared to starting serum. The range of TGF-α measured

Correspondence: H. Gregory.
Received 12 September 1988, and in revised form, 11 November 1988.

was from 10 pg to 5 ng (Figure 1) and 10 μg URO-EGF gave a reading of 40 pg TGF, i.e. same low cross reactivity as the URO-EGF assay.

The mitogenic potential of samples was assessed by observing the stimulation of radiolabelled thymidine uptake into confluent monolayers of NIH 3T3 cells in 0.5% fetal calf serum as previously described (Gregory et al., 1988). We had found that radiolabel uptake over 24 h paralleled the dose–response curves for URO-EGF and TGF-α in causing increase in actual cell numbers over 7 days. Samples were measured in triplicate.

Aliquots of 40 ml urine taken from 24 h collections from patients with advanced breast tumours and age matched controls were centrifuged at 20,000 *g* for 30 min at 4°C and then concentrated over a YM2 ultrafiltration membrane (Amicon Corporation). The concentrate (approx. 1 ml) was applied directly to a reverse phase HPLC column (Vydac C18, 25 × 0.46 cm). The elution gradient of 0.1% trifluoroacetic acid in water to 0.1% trifluoroacetic acid in acetonitrile was as defined for the separation of TGF-α and URO-EGF peptides (Gregory et al., 1988). The fractions were dried by vacuum centrifugation (Univap) and then redissolved in PBS (1 ml). Aliquots of 2 × 250 μl were taken for measurement of TGF-α by radioimmunoassay and 2 × 50 μl for URO-EGF. Accurate quantitation of total TGF-α was obtained by combining the remaining samples of fractions 43–48 and using fractions 51–56 as control. Each pooled group of urine sample was measured in duplicate (2 × 1 ml) and the relevant internal control subtracted. Processing of patient and control urines was carried out in parallel. The 24 h total (Table I) is thus given by multiplying the observed amount of TGF-α by 6/40 × 24 h urine volume.

Tumour samples obtained at surgery from a different group of patients to those providing urine samples were immediately frozen in liquid nitrogen and maintained at that temperature for longer term storage at −80°C. Receptors for oestrogen and progesterone were measured using the dextran-coated charcoal technique as described previously (Barnes et al., 1977). For both ER and PR the samples were regarded as positive if 5 or more fmol per mg of cytosol protein was measured.

The tissue samples of about 1 g were cut into small pieces and immediately blended (Ilado) for 30 s at 4°C with 5 ml buffer which comprised 20 mM Hepes, 2 mM ethylene diamine tetra acetic acid, 0.5 mM phenyl methyl sulphonyl fluoride made to pH 7.4.

The homogenate was centrifuged for 15 min at 4°C at 800 *g* and the opaque supernate spun again at 40,000 *g* for 60 min at 4°C. The residue from the latter was retained for receptor studies and the supernate diluted with two volumes of cold absolute ethanol and immediately centrifuged again at 1,250 *g*, 30 min, 4°C. The supernatant was poured into four volumes cold ethyl acetate and the mixture was kept at 4°C for 16 h. Removal of the organic phase left a very small aqueous phase which was washed with 2 ml M acetic acid solution into a small flask for lyophilysation. The dried product was taken into 0.1% aqueous trifluoroacetic acid (0.5 ml), centrifuged to remove trace insoluble materials and then applied to the reverse phase HPLC system as for the urine samples. The individual dried fractions were reconstituted in 200 μl Dulbecco minimum essential medium (DMEM) for the assays.

**Figure 1** The standard curve for the radioimmunoassay for TGF-α.

**Table I** Measurements of TGF-α and URO-EGF in urine

| | *TGF-α measured in combined HPLC fractions (ng ml$^{-1}$)* | *TGF-α total (ng 24 h$^{-1}$)* | *URO-EGF total (μg 24 h$^{-1}$)* |
|---|---|---|---|
| *Breast cancer patient* | | | |
| 1 | 1.000 | 61.5 | 3.76 |
| 2 | 0.146 | 12.9 | 3.02 |
| 3 | 0.030 | 10.1 | 9.36 |
| 4 | 0.021 | 5.3 | 5.94 |
| 5 | 0.119 | 18.1 | 5.63 |
| *Control* | | | |
| 1 | 0.001 | 0.0 | 21.08 |
| 2 | 0.059 | 26.3 | 16.58 |
| 3 | 0.050 | 13.5 | 10.57 |
| 4 | 0.086 | 14.2 | 26.22 |
| 5 | 0.028 | 7.1 | 19.07 |

MCF-7 cells were grown to a cell population of approx. $10^9$ in DMEM with 10% fetal calf serum and then maintained in serum-free medium for 3 days. The medium (1,500 ml) was filtered through a 0.45 μm filter and then concentrated over a YM2 membrane to 5 ml. This was made molar in acetic acid, centrifuged and applied to a column (100 × 1.5 cm) of BioGel P-30 (Bio-Rad) in the same solvent. Fractions (2.4 ml) were individually monitored for TGF-α by RIA. Combined lyophilysed fractions were then run on the usual HPLC system.

### Results

The TGF-α profile from MCF-7 cells (Figure 2) showed that the major product ran in the same position as the recombinant synthetic TGF-α (S-TGF-α made according to the published human structure (Marquardt et al., 1983)). Furthermore, this also ran in the S-TGF-α position in the usual HPLC system (e.g. Figure 3) after purification by affinity chromatography. The higher molecular weight component (approximately 30–40 kD) by its position on the P-30 column only, ran three or four fractions later on HPLC and as a broader peak. There was no indication of heterogeneity beyond these two components.

The detailed analytical profile for the urine of one tumour-bearing patient is shown in Figure 3 (patient no. 5, Table I). There is minimal cross-reaction between TGF-α and URO-EGF and the optical density evaluation at 206 nm (not shown) indicates sharp resolution by the HPLC column used. This was confirmed by the S-TGF-α and S-URO-EGF, subsequently used for calibration, being restricted totally to two fractions. The same column was used throughout for urine samples and gave peaks for TGF-α at fraction 44 and for URO-EGF (53 residue) at fraction 62. A second peak discernible at fraction 47 coincided with the higher molecular weight species from the MCF-7 extract.

The URO-EGF pattern was more complex and resolved normally into at least twelve definable components (unpublished data). The main components were the 53 residue protein at fraction 62, the known 52 residue protein at

Figure 2 Measurement of TGF-α by RIA in fractions from a Bio-Gel P-30 column of concentrated MCF-7 cell medium.

Figure 3 Growth factor content (per ml fraction) measured by radioimmunoassay of a concentrated urine sample – separated by HPLC, from a patient with breast cancer.

fraction 69 and a component at fraction 65 which has been only partially characterised. The latter peak occurred in all five urine samples from tumour-bearing patients but to no significant extent in the controls.

Because individual fractions gave TGF-α measurements at the less sensitive part of the RIA curve, combined samples, fractions 43–48, were used to give accurate determinations for each patient. Control measurements of fractions 51–56 varied from 6 to 26 pg ml$^{-1}$. The figures for the 10 urine samples are given in Table I. All five patients had definable TGF-α concentrations, as did four of the five controls and there was no significant difference between the groups. Total URO-EGF levels were about 1,000-fold greater than TGF-α but the amounts produced by the tumour-bearing patients were substantially lower compared with controls in every case ($P<0.005$ for the group mean values). No correlation existed between the amounts of TGF-α and URO-EGF extracted. Other studies have reported increased URO-EGF output relative to creatinine in patients with tumours (Uchihashi *et al.*, 1983) but further measurements are needed to define if these differences relate to particular types of tumour.

The tumour extracts were prepared using the solvent-based system, which in trial experiments gave 50–60% recovery of

Figure 4 Uptake of tritiated thymidine by mouse fibroblasts by HPLC fractions of the extract of a breast tumour. This tumour (0.65 g), which was negative for all three receptors, was processed as described then each fraction was reconstituted in 200 μl medium and assayed in triplicate using aliquots of 20 μl.

TGF-α and URO-EGF from starting tissue after HPLC fractionation. The final separation used the same gradient elution as for the urine samples but a different reverse phase column in which retrospective calibration with S-TGF-α and S-URO-EGF placed them at fractions 39 and 51. The profile of mitogenic activity shown by a tumour extract is given (Figure 4) with these reference points indicated. In each assay with 3T3 cells, thymidine uptake was confirmed using S-URO-EGF with a peak effect of 15–20 × background counts at 0.5–1 ng ml$^{-1}$. S-URO-EGF and S-TGF-α were of similar potency in this respect (Gregory *et al.*, 1988). In the sample shown (Figure 4), stimulatory activity was observed at the S-TGF-α position. Although the profile of mitotic activity varied widely for the tumour extracts, 4/5 samples of oestrogen (ER), progesterone (PR) receptor positive tumours, 4/5 of ER, PR negative tumours and also 4/5 of the patients pretreated with tamoxifen citrate showed detectable activity at fraction 39. The treated samples appeared to have less stimulatory capacity but this was not quantifiable in the mitogenic assays.

Because of limited amounts of each fraction, which were also used for other assays, aliquots of 20 μl from fractions 37–41 from each group of tumours were combined for accurate RIA measurement. The TGF-α content of the five ER and PR positive tumours was 14.8 ng g$^{-1}$, for the ER and PR negative tumours it was 7.4 ng g$^{-1}$ and for the ER and PR positive tumours when the patients had received tamoxifen citrate it was 0.16 ng g$^{-1}$. Although they could not be measured with statistical accuracy, the mitogenic activities of the TGF-α peaks were in keeping with the RIA quantitation.

The remaining fractions, 42 to the end, were combined in groups of five and assayed for TGF-α and URO-EGF content. None of the latter was found in any sample, particularly in the calibrated region. TGF-α immunoreactivity did extend to fractions 42–46 but it was not possible to show clearly that this was the higher molecular weight species found in MCF-7 extracts or urine samples because there was insufficient material to show stimulation in the 3T3 cell assay.

### Discussion

The presence of TGF-α in the conditioned medium of MCF-7 cells has been reported previously (Dickson *et al.*, 1986). Gel chromatography of the concentrate of serum-free medium gives two components of differing size. The higher molecular weight species appears to be of 30–40 kD which is greater than expected for a 180 amino acid precursor, whereas the lower molecular weight species synchronises on gel chromatography and subsequent HPLC systems with biosynthetic TGF-α prepared in accord with the published structure for a 50 amino acid peptide (Derynck *et al.*, 1984). Both components were measured using an RIA system with an antibody raised to the synthetic TGF-α and purified using an affinity column of the same peptide. The lower molecular weight material from MCF-7 cells, biosynthetic material and urine run as a single sharp band on an HPLC system whereas the higher molecular weight MCF-7 species appear about four fractions later as a somewhat broader band.

All samples of urine from tumour-bearing patients contained measurable TGF-α of the defined molecular species with indications that a higher molecular weight TGF-α is also present. In four of the five control samples TGF-α was measurable also and using larger amounts of control urine, not detailed here, both the 50 amino acid peptide and the higher molecular weight species could be defined. Thus, we did not see in this patient and control population the major increase in TGF-α previously reported. Nor can we agree that it is absent in control urine using immunoassay and mitogenesis as our criteria rather than receptor binding and colony forming assays which gave values of 178 $pg\,ml^{-1}$ in patient urine but undetectable values in controls (Stromberg *et al.*, 1987). The receptor assay responds to all members of the EGF family and colony formation is induced by many disparate molecules.

Some reports describe increases of URO-EGF in tumour-bearing patients (Uchihashi *et al.*, 1983). Output was related to creatinine but we have preferred to relate output to weight and we have failed to observe any increase in patients. Indeed, in the present studies all five tumour patients had a significantly lower total output than the controls. Moreover, detailed analysis of the molecular components of the mixture showed the presence of a structural variant falling between the 53 and 52 residue component on the HPLC trace which is not obvious in the controls. The relevance of this observation is as yet unclear.

Carrying out detailed extraction and measurement procedures on each tumour inevitably meant that relatively small numbers could be evaluated so group sizes of five well characterised tumours were chosen. The process devised for processing the tumour tissue was based upon knowledge of the physical properties of known agents such as URO-EGF and TGF-α. Model experiments with these two growth factors gave a final recovery of over 50% and it was thus anticipated that any small molecular factor of this family would be represented in the HPLC fractions. It was not known whether different forms, e.g. high molecular weight precursors, would also be found in the final extract. With this proviso the majority of the 15 tumours gave a peak of 'mitogenic' activity in the exact position defined by synthetic TGF-α (Figure 4). Small amounts of sample prevented dose–response curve evaluation of these samples although the stimulatory properties were less in the tamoxifen citrate-treated patient samples. This was confirmed by precise RIA evaluation of the TGF-α content of combined relevant fractions from each group. No clearly separated regions of TGF-α like immunoreactivity could be detected across the HPLC distribution nor was it possible to measure any human URO-EGF with the immunoassay which was capable of measuring down to 10 pg per sample. Whether the reduced amount of TGF-α after anti-oestrogen treatment is a simple consequence of the treatment or is directly involved in the established antitumour effect of the drug in these patients remains to be proven. In the patients with receptor negative tumours, the amounts of TGF-α did not differ substantially from the receptor positive ones. Studies are under way to observe the effect of tamoxifen citrate upon tumours in these patients.

Several groups have shown the existence of mRNA for TGF-α in both carcinomas and cell lines and the secretion of immunoreactive TGF-α in the medium from these cells (Derynk *et al.*, 1987) and, of course, the receptor is amplified in many tumour cell lines. In our studies we have found clear evidence of an active mitogen corresponding to TGF-α in the majority of breast tumours and that this is present to a reduced extent in tumours from patients treated with tamoxifen citrate. URO-EGF could not be measured in any of these samples although it is known to be present in high concentrations in all biological fluids studied (Gregory, 1985). Conversely, in the urine of both controls and patients TGF-α occurred to the same extent but the URO-EGF amounts were lower in the patients.

The mitogenic activities observed at higher fraction numbers do occur with high frequency in the extracts of all breast tumours (25 examined). They are not related immunogenically to EGF or TGF-α nor do they relate either in HPLC position or activity in our 3T3 cells with many known agents, e.g. TGF-β, IGF-1, bombesin, etc. At present, therefore, they represent novel growth factors meriting further exploration.

### References

ANZANO, M.A., ROBERTS, A.B., SMITH, J.M., SPORN, M.B. & DELARCO, J.E. (1983). Sarcoma growth factor from conditioned medium of virally transformed cells is composed of both type α and type β transforming growth factors. *Proc. Natl Acad. Sci. USA*, **80**, 6264.

BANO, M., SALOMAN, D.S. & KIDWELL, W.R. (1985). The purification of a mammary derived growth factor from human milk and human mammary tumours. *J. Biol. Chem.*, **260**, 5745.

BARNES, D., RIBEIRO, G.G. & SKINNER, L.G. (1977). Two methods for measurement of oestradiol-17-B and progesterone receptors in human breast cancer and correlation with response to treatment. *Eur. J. Cancer*, **13**, 11.

BRISSENDEN, J.E., DERYNCK, R. & FRANKE, U. (1985). Mapping of transforming growth factor α gene on human chromosome close to the breakpoint of the Burkitt's lymphoma r(2.8) variant translocation. *Cancer Res.*, **45**, 5593.

DELARCO, J.E. & TODARO, G.J. (1978). Growth factors from murine sarcoma virus-transformed cells. *Proc. Natl Acad. Sci. USA*, **75**, 4001.

DERYNCK, R., ROBERTS, A.B., WINKLER, M.E., CHEN, E.Y. & GOEDEL, D.V. (1984). Human transforming growth factor-α: Precursor and expression in *E. coli*. *Cell*, **38**, 287.

DERYNCK, R., GOEDDEL, D.V., ULLRICH, A. & 4 others (1987). Synthesis of messenger RNAs for transforming growth factors α and β and the epidermal growth factor receptor by human tumours. *Cancer Res.*, **47**, 707.

DICKSON, R.B., BATES, S.E., McMANAWAY, M.E. & LIPPMAN, M.E. (1986). Characterisation of estrogen responsive transforming activity in human breast cancer lines. *Cancer Res.*, **46**, 1707.

FRANKLIN, T.J., GREGORY, H. & MORRIS, W.P. (1986). Acceleration of wound healing by recombinant human urogastrone-epidermal growth factor. *J. Lab. Clin. Med.*, **108**, 103.

GREGORY, H. (1975). Isolation and structure of urogastrone and its relationship to epidermal growth factor. *Nature*, **257**, 325.

GREGORY, H. (1985). *In vivo* aspects of urogastrone-epidermal growth factor. *J. Cell Sci.*, Suppl. **3**, 11.

GREGORY, H., THOMAS, C.E., YOUNG, J.A., WILLSHIRE, I.R. & GARNER, A. (1988). The contribution of the C-terminal undecapeptide sequence of urogastrone-epidermal growth factor to its biological action. *Regulatory Peptides*, **22**, 217.

MARQUARDT, H., HUNKAPILLER, M.W., HOOD, L.E. & 4 others (1983). Transforming growth factors produced by petrovirus transformed rodent fibroblasts and human melanoma. *Proc. Natl Acad. Sci. USA*, **80**, 4684.

MARQUARDT, H., HUNKAPILLER, M.W., HOOD, L.E. & TODARO, G.J. (1984). Rat transforming growth factor type I: structure and relation to epidermal growth factor. *Science*, **223**, 1079.

SALOMON, D.S., ZWIEKEL, J.A., BANO, M. & 3 others (1984). Presence of transforming growth factors in human breast cancer cells. *Cancer Res.*, **44**, 4069.

SAVAGE, C.R., INAGAMI, T. & COHEN, S. (1972). The primary structure of epidermal growth factor. *J. Biol. Chem.*, **247**, 7612.

SHERWIN, S.A., TWAROZIK, D.R., BOLIN, W.H., COCKLEY, K.D. & TODARO, G.J. (1983). High molecular weight transforming growth factor activity in urine of patients with disseminated cancer. *Cancer Res.*, **43**, 403.

SPORN, M.B. & ROBERTS, A.B. (1985). Autocrine growth factors and cancer. *Nature*, **313**, 745.

STROMBERG, K., HUDGINS, W.R. & ORTH, D.N. (1987). Urinary TGFs in neoplasia: immunoreactive TGF-α in the urine of patients with disseminated breast carcinoma. *Biochem. Biophys. Res. Commun.*, **144**, 1059.

UCHIHASHI, M., HIARTA, Y., NAKAJIMA, H., FUJITA, T. & MATSUBARA, S. (1983). Urinary excretion of human epidermal growth factor (hEGF) in patients with malignant tumours. *Horm. Metab. Res.*, **15**, 261.

YEH, Y.-C., TSAI, J.-F., CHUANG, L.-Y. & 4 others (1987). Elevation of transforming growth factor α and its relationship to the epidermal growth factor and α-fetoprotein levels in patients with hepatocellular carcinoma. *Cancer Res.*, **47**, 896.